# Incidence of stroke, subsequent clinical outcomes and health care resource utilization in people with type 2 diabetes: a real-world database study in France: “INSIST” study

**DOI:** 10.1186/s12933-024-02257-4

**Published:** 2024-05-29

**Authors:** Kamel Mohammedi, Laurent Fauchier, Nadia Quignot, Artak Khachatryan, Tamar Banon, Raissa Kapnang, Kazue Kikuchi, Hongye Ren, Christine Massien, Lucile Vigié, Sara Larsen, Igor Sibon

**Affiliations:** 1 INSERM Unit 1034, Biology of Cardiovascular Diseases, Bordeaux University Hospital, 33000 Bordeaux, France; 2 Cardiology Department, Trousseau University Hospital, Tours, University of Tours, Tours, France; 3 Evidence & Access, Certara France, Paris, France; 4 Evidence & Access, Certara UK, Sheffield, UK; 5 Evidence & Access, Certara Canada, Montreal, QC Canada; 6 Novo Nordisk Denmark A/S, Copenhagen, Denmark; 7 Novo Nordisk France, Puteaux, France; 8 Stroke Unit, Bordeaux University Hospital, Bordeaux, France; 9 Department of Endocrinology, Diabetes, and Nutrition, Hôpital Haut-Lévêque, Avenue de Magellan, 33604 PESSAC CEDEX, France

**Keywords:** Type 2 diabetes, Cardiovascular disease, Atherosclerotic cardiovascular disease, Cerebrovascular disease, Stroke, Real-world study, SNDS

## Abstract

**Background:**

People with type 2 diabetes (T2D) are at elevated risk of cardiovascular disease (CVD) including stroke, yet existing real-world evidence (RWE) on the clinical and economic burden of stroke in this population is limited. The aim of this cohort study was to evaluate the clinical and economic burden of stroke among people with T2D in France.

**Methods:**

We conducted a retrospective RWE study using data from the nationally representative subset of the French Système National des Données de Santé (SNDS) database. We assessed the incidence of stroke requiring hospitalization between 2012 and 2018 among T2D patients. Subsequent clinical outcomes including CVD, stroke recurrence, and mortality were estimated overall and according to stroke subtype (ischemic versus hemorrhagic). We also examined the treatment patterns for glucose-lowering agents and CVD agents, health care resource utilization and medical costs.

**Results:**

Among 45,331 people with T2D without baseline history of stroke, 2090 (4.6%) had an incident stroke requiring hospitalization. The incidence of ischemic stroke per 1000 person-years was 4.9-times higher than hemorrhagic stroke (6.80 [95% confidence interval (CI) 6.47–7.15] versus 1.38 [1.24–1.54]). During a median follow-up of 2.4 years (interquartile range 0.6; 4.4) from date of index stroke, the rate of CVD, stroke recurrence and mortality per 1000 person-years was higher among hemorrhagic stroke patients than ischemic stroke patients (CVD 130.9 [107.7–159.0] versus 126.4 [117.2–136.4]; stroke recurrence: 86.7 [66.4–113.4] versus 66.5 [59.2–74.6]; mortality 291.5 [259.1–327.9] versus 144.1 [134.3–154.6]). These differences were not statistically significant, except for mortality (adjusted hazard ratio 1.95 [95% CI 1.66–2.92]). The proportion of patients prescribed glucagon-like peptide-1 receptor agonists increased from 4.2% at baseline to 6.6% during follow-up. The proportion of patients prescribed antihypertensives and statins only increased slightly following incident stroke (antihypertensives: 70.9% pre-stroke versus 76.7% post-stroke; statins: 24.1% pre-stroke versus 30.0% post-stroke). Overall, 68.8% of patients had a subsequent hospitalization. Median total medical costs were €12,199 (6846; 22,378).

**Conclusions:**

The high burden of stroke among people with T2D, along with the low proportion of patients receiving recommended treatments as per clinical guidelines, necessitates a strengthened and multidisciplinary approach to the CVD prevention and management in people with T2D.

**Supplementary Information:**

The online version contains supplementary material available at 10.1186/s12933-024-02257-4.

## Background

Despite improvements in the management of conventional cardiovascular risk factors [1], cardiovascular disease remains an important cause of premature morbidity and mortality among people with type 2 diabetes (T2D) [2–7]. People with T2D have a 1.5- to twofold elevated risk of stroke compared to the general population, and post-stroke outcomes [8], including mortality, are worse among people with T2D as compared to the general population without diabetes [9–12].

Newer glucose-lowering agents such as glucagon-like peptide 1 receptor agonists (GLP-1 RA) or sodium-glucose-like cotransporter 2 inhibitors (SGLT2i) have established cardiovascular disease benefits [13, 14]. Clinical guidelines, such as those from the American Diabetes Association (ADA), the European Association for the Study of Diabetes (EASD) and the European Society of Cardiology (ESC), recommend the use of these agents in people with T2D with previous cardiovascular disease or who are at high risk for cardiovascular disease [15–17]. While the use of these agents is growing, evidence indicates that they are not prescribed per guideline recommendations [6, 18, 19]. Specifically, evidence from the CAPTURE study, a cross-sectional study conducted across 13 countries, indicated that these agents were prescribed in people with T2D in 2019 at a similar rate regardless of whether the patient had previous cardiovascular disease [6]. This therefore represents a missed opportunity to prevent incident or recurrent neurological and cardiovascular events.

While the clinical and economic burden of cardiovascular disease in people with T2D has been previously described using national databases in Europe [20], less is known about post-stroke clinical outcomes, management practices and costs in the T2D population in real-world settings. The French national health database, the Système National des Données de Santé (SNDS), which captures claims data for 99% of the French population ($\sim 66$ million), offers unique opportunities to examine treatment practices, clinical outcomes, health care resource utilization and costs following incident stroke in a nationally representative sample of people with T2D. The overall objective of the present study was to estimate the clinical and economic burden of stroke among people with T2D in France.

## Methods

### Study population

Data for this retrospective cohort study were extracted from the *Echantillon Général des Bénéficiaires* (EGB), a random sample of the SNDS database including approximately 700,000 people. Data from this source are linked via unique social security numbers to primary care, hospital, pharmacy, and death registration databases.

The study population included adults (age > 18 years) with T2D who had an incident stroke requiring hospitalization between 1 January 2012 and 31 December 2018. Index date was the date of stroke hospital admission, and baseline was the 24 months prior to index date. Patients were followed from index date until the earliest of 31 December 2019 or date of death (Additional file 1: Fig. S1). All patients excluding those that died were followed for a minimum of 12 months.

People with T2D were identified either using the International Classification of Disease, version 10 (ICD-10) code E11 in the long-term disease database or from a hospital discharge diagnosis, or using at least three records for reimbursements of oral antidiabetics or insulin. People with both a diagnostic record for type 1 diabetes (ICD-10: E10) and insulin monotherapy were excluded. Incident stroke events were identified with relevant ICD-10 diagnostic codes (ischemic stroke: I63 or G45 [principal diagnosis], combination of G46 [principal diagnosis] + I63 [secondary diagnostic]; hemorrhagic stroke: I60-I62 [principal diagnosis], combination of G46 [principal diagnosis] + I60-I62 [secondary diagnosis]; unspecified stroke: I64 [principal diagnosis], combination of G46 [principal diagnosis] + I64 [secondary diagnosis]). Patients with any prior history of stroke were excluded.

### Study variables and definitions

Patient baseline demographics (age and sex) and clinical characteristics including time from first evidence of T2D to first stroke, comorbidities, Charlson Comorbidity index [21], diabetes treatments (biguanides, sulfonylureas, insulin, dipeptidyl peptidase-4 inhibitor [DPP-4 inhibitors], meglitinides, alpha-glucosidase inhibitors, thiazolidinediones, GLP-1 RA), and cardiovascular disease treatments (anti-hypertensives, anticoagulants, antiplatelets, statins) were collected during the baseline period. SGLT2i were excluded from the list of diabetes treatments since they had not entered the French market during the study period. The GLP-1 RA exenatide and liraglutide entered the French market in 2008 and 2009, respectively, while dulaglutide did not enter until 2016. As such, usage of GLP-1 RA reported in this study represents early uptake. Full definitions of all variables are presented in Additional file 1: Table S1.

### Clinical outcomes

Stroke recurrence was defined based on ICD-10 diagnostic codes and was assessed from index date + 21 days until date of stroke recurrence, death or end of follow-up [22]. Development of cardiovascular diseases, defined as coronary artery disease, cardiac arrhythmias, cerebrovascular disease (including stroke), peripheral arterial disease (PAD), heart failure or aortic disease, as well as thromboembolic events, were defined based upon the identification of relevant codes during the follow-up period. All-cause mortality was assessed from index date until end of follow-up.

### Treatments

Diabetes and cardiovascular disease treatments administered during follow-up were captured and presented among patients who did not die without the corresponding treatments recorded.

### Health care resource utilization (HCRU)

Patient HCRU during follow-up was captured under the following categories: index stroke hospitalization, subsequent hospitalizations (day and overnight stay cases/overnight stays only/intensive care unit stays), outpatient physician visits according to specialty, physical rehabilitation therapy visits.

### Costs

Annual costs of HCRU per patient were estimated in Euros during follow-up, including index date. Costs included all costs incurred within hospital, external consultation costs and outpatient costs.

#### Statistical analysis

The crude incidence of stroke among people with T2D in the study period was estimated per 1000 person-years. The corresponding two-sided 95% confidence interval (CI) was calculated using the Poisson distribution. Directly age-standardized incidence rates were calculated using the French population as the reference population. Baseline and outcome variables were described overall and per stroke subtype using descriptive statistics. Differences in baseline characteristics between ischemic stroke patients and hemorrhagic stroke patients were assessed using Mann–Whitney tests or t-tests for continuous variables, and with Chi-square tests for categorical variables; where more than 25% of the cells exhibited expected counts below 5, the Fisher Exact test was applied. Incidence of clinical and HCRU outcomes were estimated per 1000 person-years of follow-up with corresponding 95% CIs. Differences in time to event outcomes between ischemic stroke and hemorrhagic stroke patients were assessed using Kaplan–Meier curves and compared using log-rank tests. We also used Cox proportional hazards models to estimate hazard ratios (HRs) and 95% CIs for the incidence of outcomes in patients with ischemic vs. hemorrhagic stroke, after adjusting for age, sex and baseline history of arrythmia (which was significantly associated with stroke subtypes at baseline).

## Results

### Incidence of stroke

Overall, data for 45,331 people with T2D were extracted (Additional file 1: Fig. S2). Of these, 2090 (4.6%) people had an incident stroke between 2012 and 2018. This equated to an overall crude stroke incidence rate of 9.0 per 1000 person-years. Age-standardized incidence rates of stroke declined from 10.3 per 1000 person-year in 2012 to 7.6 per 1000 person-year in 2018.

Of the 2090 incident strokes, 1582 (75.7%) were ischemic strokes, 325 (15.6%) were hemorrhagic strokes, and 183 (8.6%) were unspecified stroke. The incidence of ischemic stroke was 4.9-times higher than that of hemorrhagic stroke (6.8 versus 1.4 per 1000 person-years).

### Baseline characteristics

Baseline characteristics of the 2090 incident stroke patients are presented in Table 1, overall and according to stroke subtype; results on unspecified stroke are not presented. The mean (standard deviation [SD]) age of the study population was 75.0 (11.4) years, and 1156 (55.3%) were male. The most common comorbid conditions among patients included hypertension (83.0%), dyslipidemia (63.6%) and cardiac arrhythmias (19.4%).

**Table 1:** Baseline characteristics of people with T2D with stroke hospitalization, overall and by type of stroke

	All	Ischemic stroke	Hemorrhagic stroke	P value
N (%)	2090 (4.6%)	1582 (75.7%)	325 (15.6%)	
Mean (SD) age at index	75.0 (11.4)	75.3 (11.3)	74.6 (2.0)	0.5384
Sex, n (%)				
Male	1156 (55.3)	846 (53.5)	200 (61.5)	0.0078
Female	934 (44.7)	736 (46.5)	125 (38.5)	
Median (IQR) time from first evidence of T2D to first stroke, years	6.8 (3.5; 12.3)	6.8 (3.4; 12.3)	6.4 (3.5; 12.3)	0.9633
Comorbidities, n, (%)				
Cardiac arrhythmias	405 (19.4)	292 (18.5)	76 (23.4)	0.0404
Coronary heart disease	317 (15.2)	237 (15.0)	47 (14.5)	0.8106
Hypertension	1,734 (83.0)	1,319 (83.4)	263 (80.9)	0.2842
Dyslipidemia	1,329 (63.6)	1,001 (63.3)	200 (61.5)	0.5550
Obesity	249 (11.9)	187 (11.8)	35 (10.8)	0.5904
Atherosclerosis	236 (11.3)	172 (10.9)	35 (10.8)	0.9566
Chronic heart failure	240 (11.5)	172 (10.9)	35 (10.8)	0.9566
Heart failure	238 (11.4)	170 (10.8)	35 (10.8)	0.9901
Myocardial infarction	292 (14.0)	218 (13.8)	42 (12.9)	0.6818
All type of cerebrovascular disease (excluding stroke)	221 (10.6)	171 (10.8)	23 (7.1)	0.0426
Peripheral artery disease	178 (8.5)	136 (8.6)	24 (7.4)	0.4728
Chronic kidney disease and renal failure	245 (11.7)	166 (10.5)	44 (13.5)	0.1102
Acute renal failure	96 (4.6)	66 (4.2)	14 (4.3)	0.9115
Renal failure	140 (6.7)	96 (6.1)	19 (5.9)	0.8782
Chronic kidney disease	201 (9.6)	136 (8.6)	35 (10.8)	0.2118
Pulmonary embolism	451 (21.6)	328 (20.7)	68 (20.9)	0.9387
Deep vein thrombosis	274 (13.1)	214 (13.5)	36 (11.1)	0.2334
Age-adjusted Charlson Comorbidity Index, n (%)				0.6679
0 points	11 (0.5)	10 (0.6)	Freq < 10	
1–2 points	155 (7.4)	116 (7.3)	28 (8.6)	
3–4 points	678 (32.4)	522 (3.0)	100 (30.87)	
≥ 5 points	1246 (59.6)	934 (59.0)	196 (60.3)	

Results on unspecified stroke are not presented, ischemic stroke and hemorrhagic stroke do not sum up to all stroke

*IQR* interquartile range, *SD* standard deviation, *TD2* type 2 diabetes

### Clinical outcomes

**Fig. 1:**
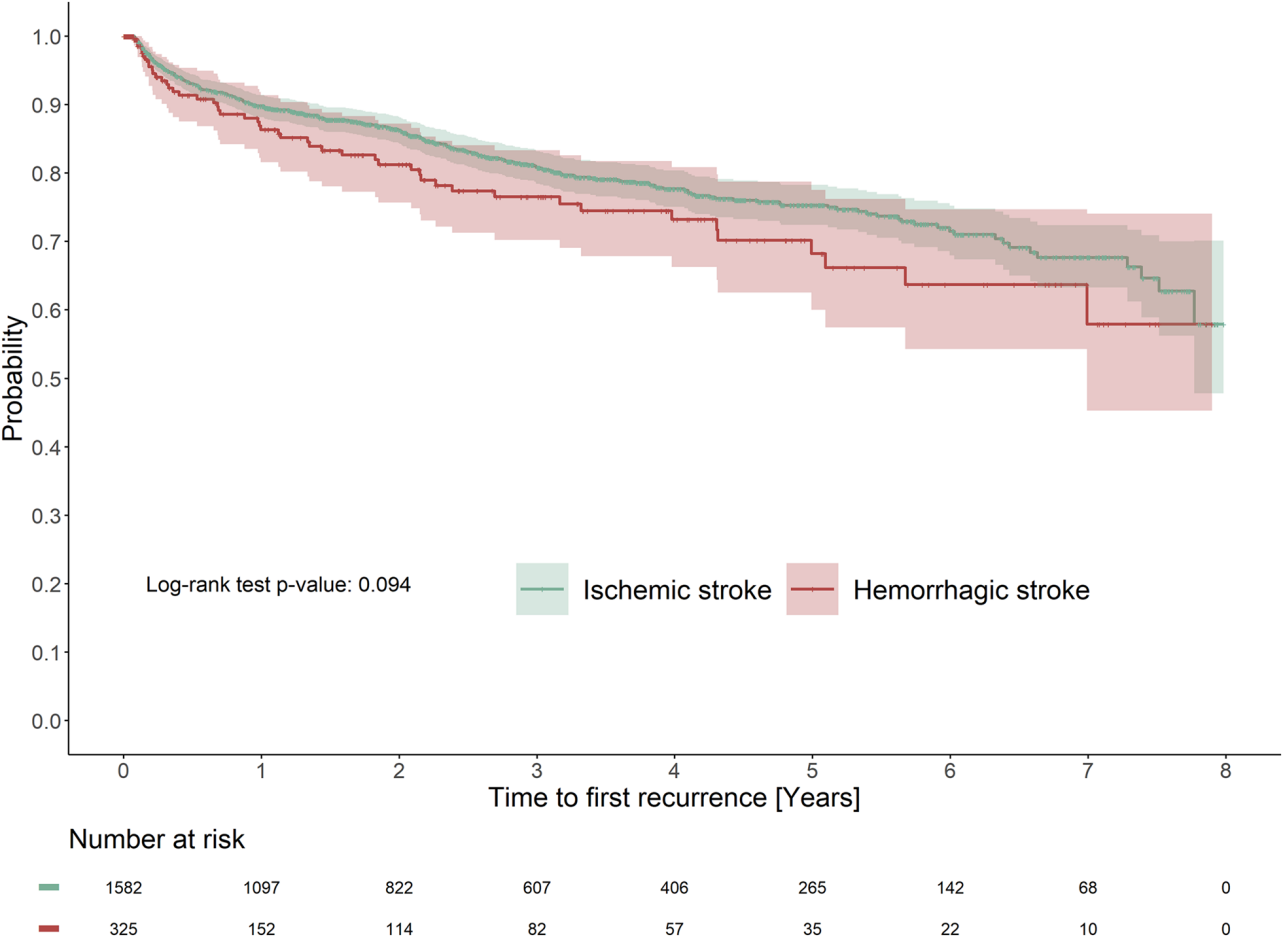
Time to first stroke recurrence according to incident stroke subtype

Overall, the 1-year cumulative incidence of stroke recurrence was 10.7% (95% CI 9.2–12.2), with no significant difference when stratified by subtype of index stroke (*p* = 0.094) (Fig. 1). During a mean follow-up of 2.8 years from index stroke, 53.2% of patients (55.9% versus 38.5% of ischemic and hemorrhagic stroke patients, respectively) were hospitalized for cardiovascular disease (Table 2). The most common cardiovascular disease outcomes were cardiac arrhythmias, coronary heart disease (CHD), and heart failure, with rates of 106.2 (95% CI 97.8–115.3), 69.8 (63.5–76.7) and 69.3 (62.7–76.6) per 1000 person-years, respectively. There was no significant difference in risk of any subsequent cardiovascular disease according to stroke subtype after adjustment (Table 2). Time to first cardiovascular event for each clinical outcome and according to index stroke subtype is presented in Additional file 1: Fig. S3.

**Table 2:** Clinical outcomes during follow-up, overall and by type of stroke

	All	Ischemic stroke	Hemorrhagic stroke	P value
N	2090	1582	325	
Follow-up time				
Mean (SD)	2.8 (2.3)	2.9 (2.2)	2.1 (2.3)	
Median (IQR)	2.4 (0.6; 4.4)	2.6 (1.0; 4.5)	1.2 (0.0; 4.0)	
Clinical Outcomes				
Cardiovascular disease outcomes				
Any cardiovascular disease outcome after discharge from index stroke hospitalization, n (%)	1112 (53.2)	884 (55.9)	125 (38.5)	< 0.0001
Rate per 1000 person-years (95% CI)	356.1 (339.6–373.3)	353.3 (335.1–372.6)	338.5 (293.6–390.4)	
Adjusted^a^ hazard ratio (95% CI)		1 (reference)	0.94 (0.78–1.13)	0.5094
All type of cerebrovascular disease, n (%)	733 (35.1)	581 (36.7)	88 (27.1)	0.0009
Rate per 1000 person-years (95% CI)	126.9 (118.6–135.8)	126.43 (117.2–136.4)	130.9 (107.7–159.0)	
Adjusted^a^ hazard ratio (95% CI)		1 (reference)	1.01 (0.81–1.27)	0.9219
Cardiac arrhythmia, n (%)	507 (24.3)	410 (25.9)	55 (16.9)	0.0006
Rate per 1000 person-years (95% CI)	106.2 (97.8–115.3)	108.93 (99.4–119.4)	94.7 (73.6–121.7)	
Adjusted^a^ hazard ratio (95% CI)		1 (reference)	0.97 (0.73–1.29)	0.8295
Coronary heart disease, n (%)	403 (19.3)	331 (20.9)	30 (9.2)	< 0.0001
Rate per 1000 person-years (95% CI)	69.8 (63.5–76.7)	72.03 (64.9–79.9)	44.6 (31.5–63.3)	
Adjusted^a^ hazard ratio (95% CI)		1 (reference)	0.95 (0.65–1.40)	0.8057
Heart Failure, n (%)	357 (17.1)	287 (18.1)	34 (10.5)	0.0008
Rate per 1000 person-years (95% CI)	69.3 (62.7–76.6)	70.40 (63.0–78.7)	53.97 (38.9–74.8)	
Adjusted^a^ hazard ratio (95% CI)		1 (reference)	0.92 (0.64–1.32)	0.6433
Peripheral arterial disease, n (%)	230 (11.0)	182 (11.5)	18 (5.5)	0.0014
Rate per 1000 person-years (95% CI)	43.0 (37.9–48.8)	42.7 (37.1–49.3)	28.4 (18.0- 44.8)	
Adjusted^a^ hazard ratio (95% CI)		1 (reference)	0.88 (0.53–1.46)	0.6178
Carotid artery disease, n (%)	115 (5.5)	100 (6.3)	Freq < 10	
Rate per 1000 person-years (95% CI)	19.9 (16.6–23.9)	21.76 (17.9–26.4)	8.9 (4.0–19.8)	
Adjusted^a^ hazard ratio (95% CI)		1 (reference)	0.48 (0.19–1.21)	0.1196
Aortic disease, n (%)	52 (2.5)	41 (2.6)	Freq < 10	
Rate per 1000 person-years (95% CI)	9.0 (6.9–11.8)	8.9 (6.6- 12.1)	4.5 (1.4- 13.8)	
Adjusted^a^ hazard ratio (95% CI)		1 (reference)	0.51 (0.11–2.32)	0.3808
Stroke recurrence				
Stroke recurrence following index stroke hospitalization, n (%)	344 (16.5)	269 (17.0)	49 (15.1)	0.3961
Rate per 1000 person-years (95% CI)	68.2 (61.6–75.5)	66.5 (59.2–74.6)	86.7 (66.4–113.4)	
Adjusted^a^ hazard ratio (95% CI)		1 (reference)	1.06 (0.78–1.44)	0.7264
Thromboembolism				
Pulmonary embolism, n (%)	512 (24.5)	404 (25.5)	63 (19.4)	0.0099
Rate per 1000 person-years (95% CI)	111.0 (102.3–120.5)	109.5 (99.8- 120.0)	121.5 (96.4–153.1)	
Adjusted^a^ hazard ratio (95% CI)		1 (reference)	1.17 (0.90–1.53)	0.2387
Deep vein thrombosis, n (%)	329 (15.7)	255 (16.1)	45 (13.9)	0.2542
Rate per 1000 person-years (95% CI)	65.8 (59.3–73.0)	63.6 (56.5- 71.6)	79.7 (60.2- 105.5)	
Adjusted^a^ hazard ratio (95% CI)		1 (reference)	1.07 (0.78–1.48)	0.6713
Mortality				
N (%)	952 (45.6)	662 (41.9)	196 (60.3)	< 0.0001
30-day mortality, n (%)	341 (16.3)	196 (12.4)	115 (35.4)	< 0.0001
6-month mortality, n (%)	504 (24.1)	312 (19.7)	143 (44.0)	< 0.0001
1-year mortality, n (%)	591 (28.3)	378 (23.9)	153 (47.1)	< 0.0001
Mortality rate per 1000 person-years	164.9 (155.6–174.7)	144.1 (134.3- 154.6)	291.5 (259.1- 327.9)	
Adjusted^a^ hazard ratio (95% CI)		1 (reference)	1.95 (1.66–2.92)	< 0.0001

Results on unspecified stroke are not presented, ischemic stroke and hemorrhagic stroke do not sum up to all stroke

*CI* Confidence intervals, *IQR* interquartile range, *SD* standard deviation

^a^Adjusted for age, sex and baseline history of arrythmia

**Fig. 2:**
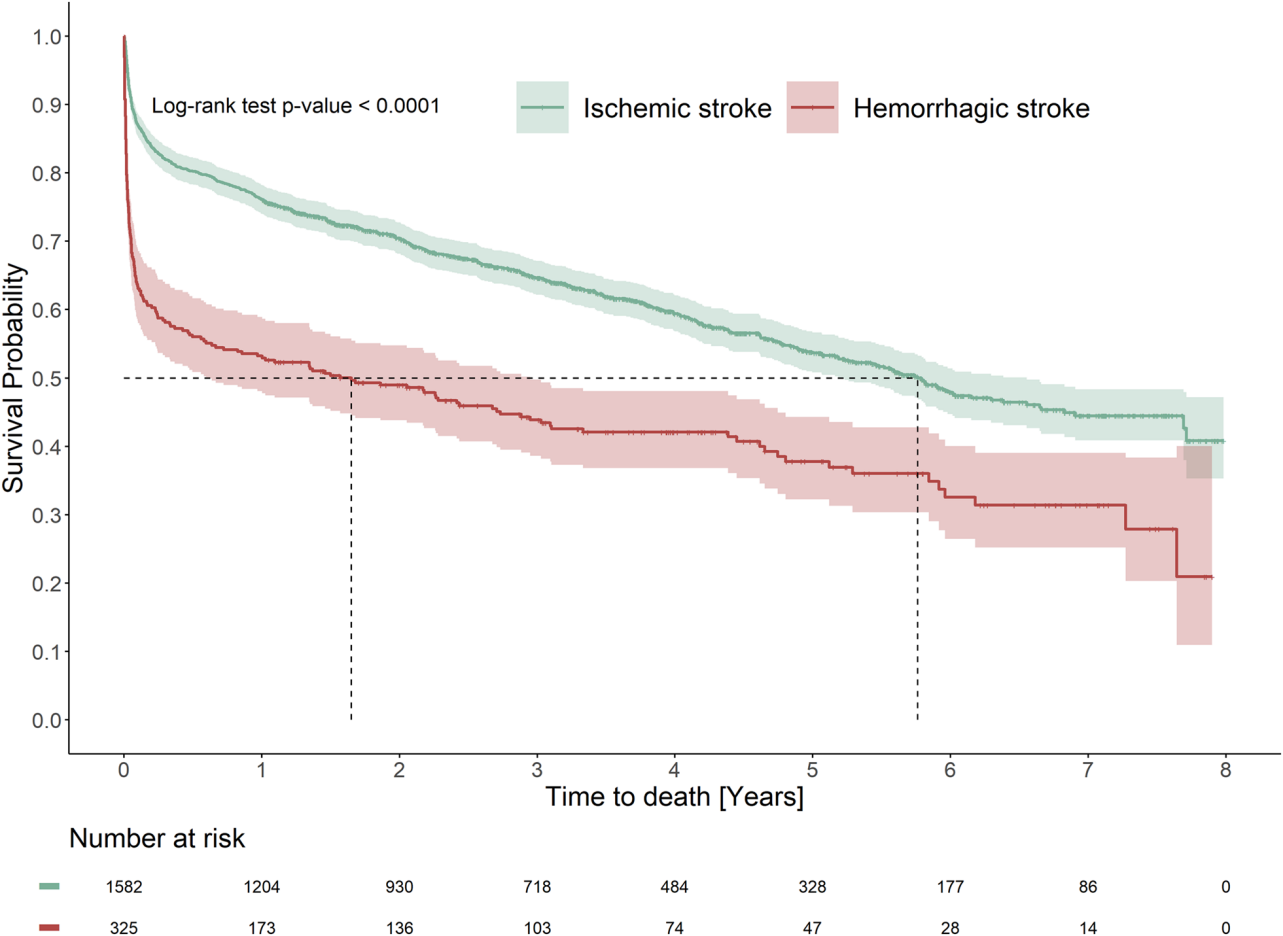
Time to death according to incident stroke subtype

The overall mortality rate was 164.9 (155.6–174.7) per 1,000 person-years, and risk of mortality was significantly higher among hemorrhagic stroke patients than ischemic stroke patients (adjusted HR [95% CI] 1.95 [1.66–2.92]) (Fig. 2, Table 2). Overall, the 30-day and one-year mortality rates were 16.3% and 28.3%, respectively. Hemorrhagic stroke mortality rate at 30-days was 23 percentage points higher than for ischemic stroke (Table 2).

### Treatment patterns

**Fig. 3:**
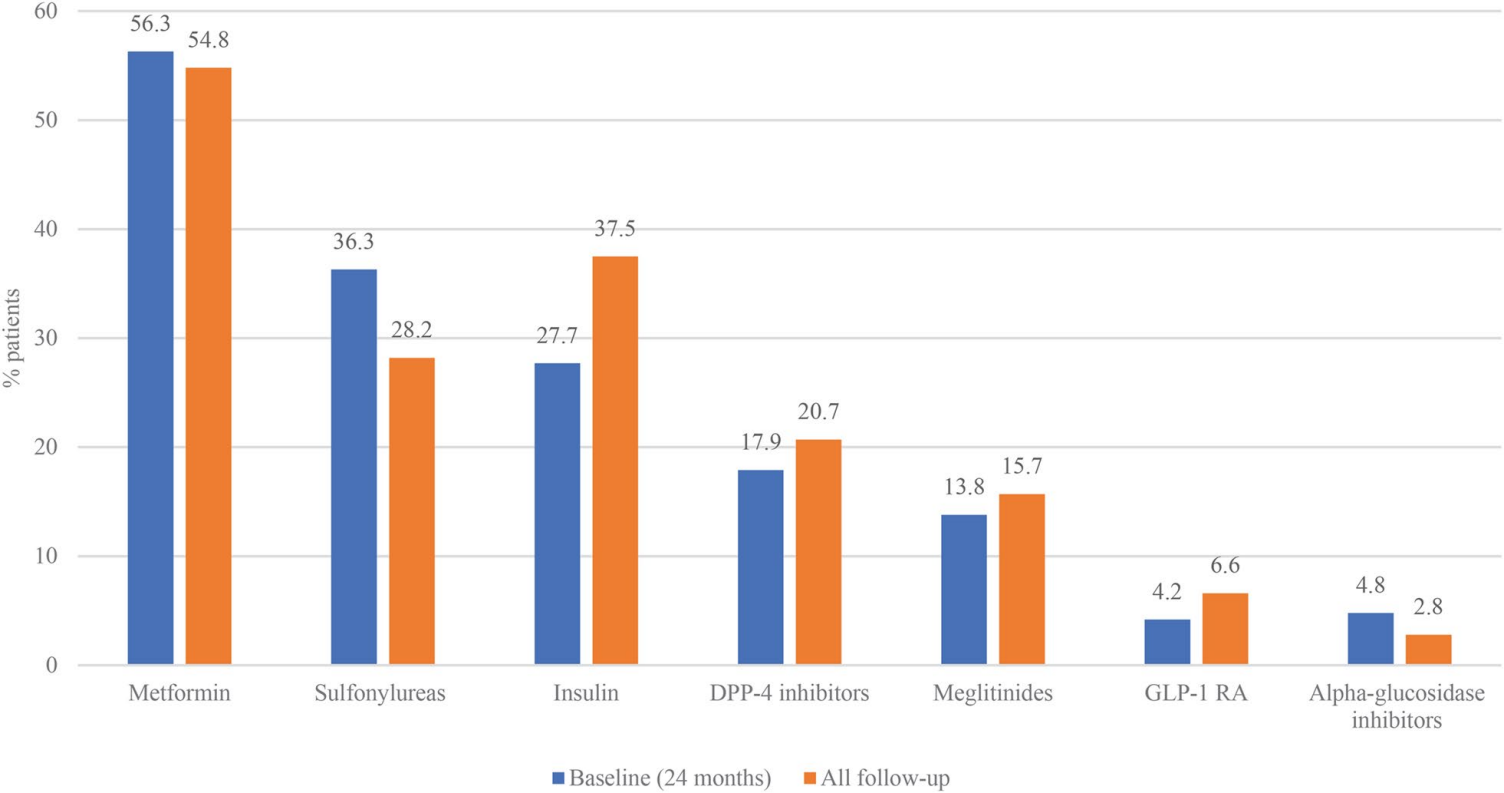
Use of diabetes treatment before and after index stroke hospitalization. *DPP-4* dipeptidyl peptidase, *4 GLP-1* Glucagon-like peptide-1, *PAD* peripheral arterial disease, *RA* receptor agonist

**Fig. 4:**
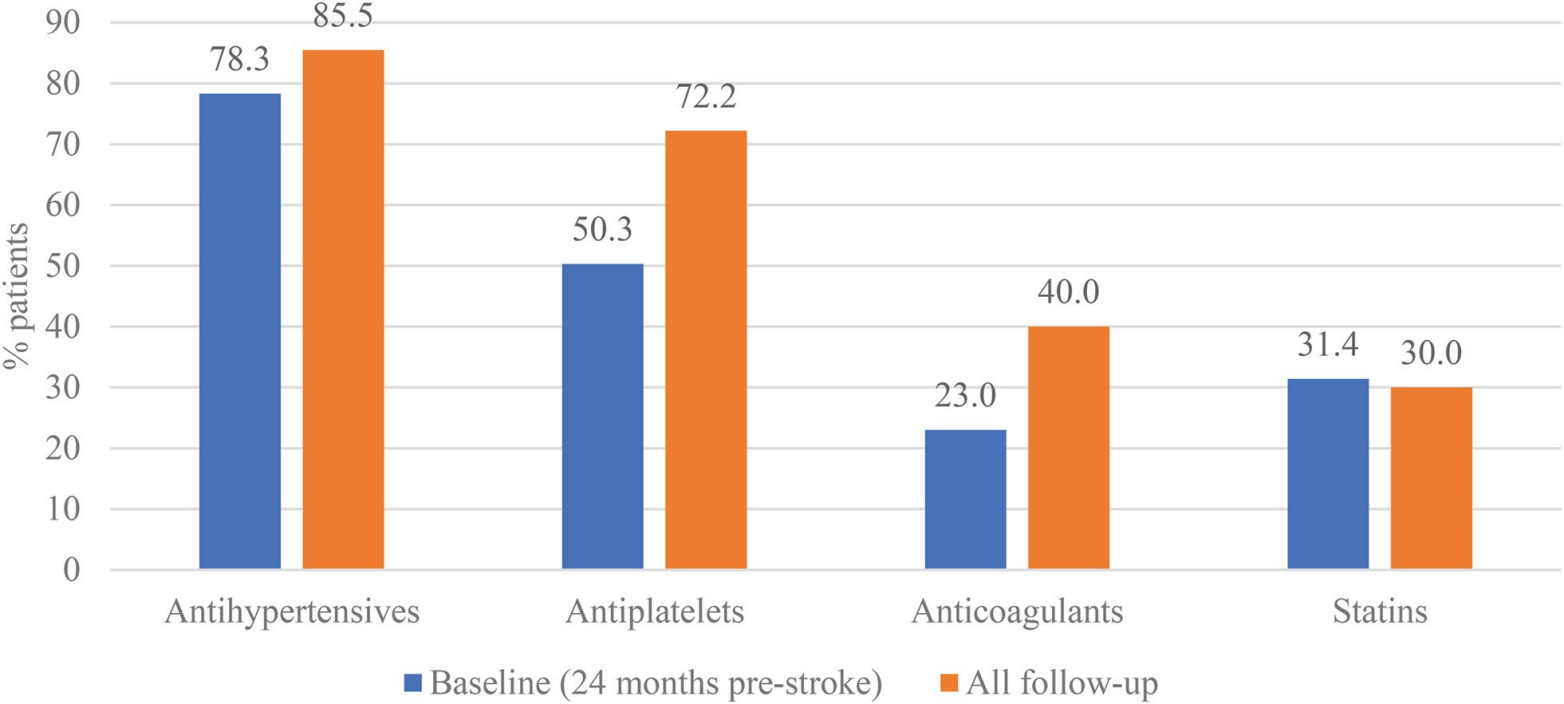
Use of cardiovascular treatment before and after index stroke hospitalization

**Fig. 5:**
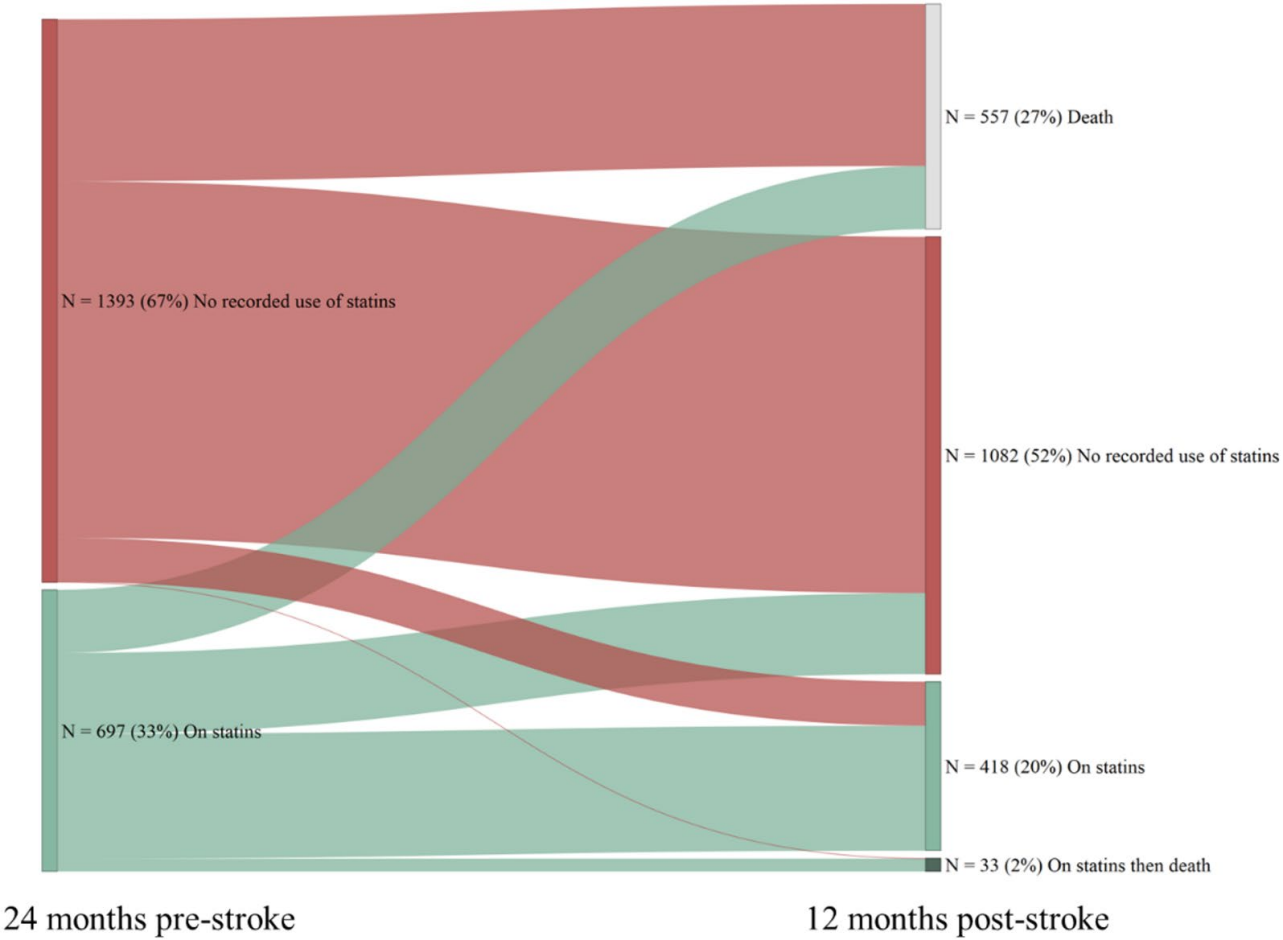
Statins use before and after index stroke hospitalization

Metformin was the most prescribed glucose-lowering agent, used in 56.3% patients pre-stroke and 54.8% patients during follow-up (Fig. 3). The use of insulin increased from 27.7% pre-stroke to 37.5% during follow-up. The use of GLP-1 RA increased from 4.2% pre-stroke to 6.6% during follow-up. The use of most cardiovascular therapies increased following incident stroke (Fig. 4). In the 24 months prior to stroke, 78.3% of patients were prescribed anti-hypertensive medications and this increased to 85.5% during follow-up. The proportion of patients prescribed statins following the index stroke remained largely unchanged compared to the previous 24-months (30.0% versus 31.4%) (Fig. 4), with only a small proportion (8%) of untreated patients switching to statins during the first 12 months following the index stroke (Fig. 5). Treatment patterns trends were similar for patients with index ischemic and hemorrhagic stroke.

### Health care resource utilization

Approximately two thirds of the cohort (68.8%) had a subsequent hospitalization following the index stroke hospitalization (Table 3). The rate of subsequent hospitalizations was 2234 per 1000 person-years overall and was considerably higher in hemorrhagic stroke patients than in ischemic stroke patients (hemorrhagic stroke: 3110 per 1000 person-years, ischemic stroke:1926 per 1000 person-years). Among the 63.7% of the cohort who had a subsequent overnight hospitalization, the median (IQR) length of stay was 7.5 (5.0–11.3) days; it was similar according to stroke subtype.

**Table 3:** Health care resource use and costs in participants, overall and by type of stroke

	All	Ischemic stroke	Hemorrhagic stroke	P value
N	2090	1582	325	
Follow-up time				
Mean (SD), years	2.8 (2.3)	2.9 (2.2)	2.1 (2.3)	
Median (IQR), years	2.4 (0.6; 4.4)	2.6 (1.0; 4.5)	1.2 (0.0; 4.0)	
Hospitalizations				
Subsequent overall hospitalization (day and overnight stays) following index stroke hospitalization, n (%)	1437 (68.8)	1135 (71.7)	168 (51.7)	< 0.0001
Rate per 1000 person-years (95% CI)	2234 (2192–2278)	1926 (1888–1965)	3110 (2922–3309)	
Subsequent overnight hospitalization following index hospitalization, n (%)	1332 (63.7)	1050 (66.4)	157 (48.3)	< 0.0001
Rate per 1000 person-years (95% CI)	1041 (1035–1046)	960 (955–966)	1153 (1121–1185)	
Subsequent stay in ICU, n (%)	260 (12.4)	217 (13.7)	21 (6.5)	0.0003
Rate per 1000 person-years (95% CI)	66 (6–73)	68 (61–76)	52 (38–72)	
Outpatient visits				
At least 1 outpatient visit, n (%)	1708 (81.7)	1353 (85.5)	206 (63.4)	< 0.0001
Rate per 1000 person-years (95% CI)	9213 (8991–9440)	8929 (8689–9175)	9405 (8756–10,102)	
Outpatient visits to a General Practitioner, n (%)	1439 (68.9)	1143 (72.3)	169 (52.0)	< 0.0001
Rate per 1000 person-years (95% CI)	5124 (5006–5244)	5148 (5006–5283)	5233 (4889–5602)	
Outpatient visits to a neurologist, n (%)	512 (24.5)	396 (25.0)	89 (27.4)	0.3751
Rate per 1000 person-years (95% CI)	191 (181–201)	179 (169–191)	323 (289–360)	
Other outpatient specialties, n (%)				
Cardiovascular pathology	655 (31.3)	541 (34.2)	58 (17.9)	< 0.0001
Endocrinology and metabolism	322 (15.4)	262 (16.6)	31 (9.5)	0.0014
Rehabilitation				
Any rehabilitation visit (speech or physical rehabilitation), n (%)	1140 (54.6)	900 (56.9)	141 (43.4)	< 0.0001
Rate per 1000 person-years (95% CI)	12,404 (12,101–12,715)	12,168 (11,836–12,510)	14,719 (13,683–15,833)	
Costs, euros				
Yearly hospital admission costs (including index stroke admission)				
Mean (SD)	9673 (12,905)	8812 (10,921)	11,462 (16,385)	
Median (IQR)	5683 (2634–11,292)	5375 (2485–10,627)	6727 (3366–12,080)	
Yearly cost of external consultation				
Mean (SD)	434 (716)	441 (726)	384 (685)	
Median (IQR)	183 (0–545)	199 (15–553)	72 (0–412)	
Yearly cost of outpatient consumption				
Mean (SD)	8485 (11,267)	8374 (10,116)	7610 (13,345)	
Median (IQR)	4890 (1599–11,316)	5150 (1928–11,315)	3282 (109–10,141)	
Overall total yearly costs				
Mean (SD)	18,592 (20,558)	17,627 (17,548)	19,456 (25,916)	
Median (IQR)	12,199 (6846–22,378)	11,947 (6851–21,764)	11,985 (6236–21,753)	

Results on unspecified stroke are not presented, ischemic stroke and hemorrhagic stroke do not sum up to all stroke

*CI* Confidence intervals, *ICU* intensive care unit, *IQR* interquartile range, *SD* standard deviation

During follow-up, 81.7% of patients had a record of outpatient visits with a greater proportion of ischemic stroke (85.5%) patients having outpatient visits than hemorrhagic stroke patients (63.4%). The overall rate of outpatient visits was 9213 per 1000 person-years. The most common outpatient visits were to general practitioners (68.9%) and cardiovascular specialists (31.3%). Stroke rehabilitation visits occurred in more than half the cohort during follow-up (54.6%) at a rate of 12,404 per 1000 person-years.

#### Costs

Overall, the median (IQR) yearly hospital admission costs for stroke patients with T2D, including the cost of all hospitalizations (including index hospitalization), procedures, and drugs administered in hospitals was €5683 (2634–11,292). Median yearly hospital admission costs were higher for hemorrhagic stroke patients than ischemic stroke patients: €6727 (3366–12,080) versus €5,375 (2485–10,627), respectively. The median (IQR) yearly outpatient costs, including cost of visits and consultations, drugs, and procedures outside of hospitals was €4890 (1599–11,316). The median (IQR) overall total yearly cost incorporating hospital admissions, external consultations, and all outpatient costs was €12,199 (6846–22,378), with no significant difference by index stroke subtype (ischemic stroke: 11,947 [6851–21,764], hemorrhagic stroke: 11,985 [6236–21,753]).

## Discussion

### Key findings

In this real-world study using data representative of the overall French population, the incidence of stroke was high, affecting 4.6% of the people with T2D. Of the incident strokes, 75.7% were ischemic and 15.6% were hemorrhagic. The overall clinical and economic burden of stroke was substantial, with a 1-year cumulative incidence of stroke recurrence of 10.7%, 1-year mortality rate of 28.3%, and a median total yearly cost of €12,199. Risk of mortality was significantly higher following hemorrhagic stroke than ischemic stroke (adjusted HR: 1.95 [1.66–2.92]), though there was no significant difference in subsequent cardiovascular disease outcomes by stroke subtype.

### Relation to previous studies

The observed overall age-standardized incidence rate of stroke of 9.0 per 1000 person-year during the study period aligns closely with data from the Swedish National Diabetes Register for the period 1998–2012 in which incidence rate of stroke among people with T2D was 8.8 per 1000 person-years [2]. Similarly, data from Australia indicated a crude incidence rate for hospitalization for stroke of 6.5 per 1000 person-years among people with T2D in 2018 [23]. Disparities in incidence rates between earlier data and the present study likely reflect key differences in the distribution and management of cardiovascular risk factors between the countries and over time. The decline in incidence of stroke in the present study between 2012 and 2018 illustrates the likely impact of improvements in cardiovascular disease risk factor management. Nonetheless, the incidence of stroke among patients with T2D remains considerably higher than observed in the general population. Data from the Global Burden of Disease collaborators indicated that the overall age-standardized incidence rate of stroke was 1.5 (1.4–1.7) per 1000 person-year in 2019 among the general population with or without diabetes [24].

The Incidence rate for ischemic stroke reported in the present study (6.8 per 1000 person-year) also aligns closely with findings from national registries in Scotland [9]. In contrast, findings from Sweden indicate an ischemic stroke incidence rate of 3.7 per 1000 person-years in 2013, a possible reflection of difference in distribution and management of cardiovascular disease risk factors between French and Swedish populations [9, 25].

These data highlight that while there have been reductions in the incidence of cardiovascular outcomes such as stroke over time, people with T2D remain at an elevated risk of developing stroke [26, 27].

There are limited contemporary data on post-stroke outcomes for T2D populations. However, most evidence indicates that people with T2D are at elevated risk of adverse outcomes following stroke compared to people without T2D [2, 11, 28, 29]. The burden of cardiovascular disease following stroke in the present study was considerable. Cardiac arrhythmias were the most common cardiovascular outcome following incident stroke, with an overall rate of 106.2 (97.8–115.3) per 1000 person-years.

The proportion of patients receiving anticoagulation therapy during follow-up (40.0%) surpassed the proportion of patients with cardiac arrhythmias (24.3%). This finding likely reflects the use of anticoagulants for the prevention and/or treatment of other indications such as thromboembolic diseases. For example, 24.5% of patients had evidence of pulmonary embolism during follow-up. Another potential explanation might be the potential under-estimation of cardiac arrhythmias due to the use of hospital discharge diagnosis codes to detect this outcome. The probability of atrial fibrillation following ischemic stroke has been demonstrated to be high, regardless of whether the stroke can be attributed to other non-cardioembolic causes such as large-artery atherosclerotic disease or small-vessel occlusive disease [30–32]. Given that atrial fibrillation increases the risk of ischemic stroke five-fold [33], the findings highlight the importance of accurate cardiac arrythmia evaluation and subsequent antithrombotic therapy in stroke patients with T2D. Though not statistically significant, the incidence of cardiac arrythmias following stroke was also higher among ischemic stroke than hemorrhagic stroke patients (108.93 [95% CI 99.4–119.4] vs. 94.7 [73.6–121.7]). This finding has been demonstrated in previous studies conducted among the general population [34]. For example, in a US-based study ischemic stroke patients had a higher rate of death due to arrhythmia than hemorrhagic stroke patients (7.3% vs. 18.7%) [34]. The incidence of coronary heart disease and carotid artery disease was also higher among ischemic stroke than hemorrhagic stroke in the current study. In a study undertaken using the UK Clinical Practice Research Datalink, rates of coronary heart disease were slightly higher among ischemic stroke than hemorrhagic stroke patients (1.19 per 100 person-years vs. 1.07 per 100 person-years), though this difference was not significant [35].

In people with established cardiovascular disease or at high risk for cardiovascular disease, GLP-1 RA and SGLT2i have been recommended by the consensus reports of the ADA and the EASD since 2018 [36]. This recommendation was based upon established cardiovascular disease benefit as demonstrated in clinical trials and observational studies [13, 14, 37–39]. That so few patients were subsequently prescribed GLP-1 RA following incident stroke in the present study (6.6%) likely reflects the lack of recommendations for the use of these treatments to prevent cardiovascular disease in most of the years covered by this study [6]. Nonetheless, the CAPTURE study demonstrated that patients with previous cardiovascular disease were slightly less likely to be prescribed GLP-1 RA than patients without previous cardiovascular disease (9.5% versus 10.4%) [6]. Similar findings were observed in the SWEDEHEART study [40]. Treatment patterns studies have also demonstrated low utilization of these drugs, including among patients with established cardiovascular disease [41, 42]. In the present study, even the utilization of statins remained very low (30.0%) following stroke, despite clinical guidelines for cardiovascular disease management in T2D populations strongly recommending the use of these agents among people at high risk of cardiovascular disease [17, 43]. Stronger adherence to clinical guidelines through enhanced educational activities remains a key priority to expand the use of cardioprotective drugs in this high-risk population. Furthermore, given that there is some evidence to indicate that glycemic control may be associated with stroke severity [44], improved management of hyperglycemia may have beneficial impacts on the incidence and severity of stroke.

This study has also demonstrated the considerable economic burden associated with stroke in T2D populations. The estimated total yearly medical median costs for people with T2D with stroke was €12,199 (6846–22,378) in the present study. This represents a larger estimate than observed in Scotland for incident cerebrovascular disease for the period 2015–2016 (mean annual costs: £7900 [1900–32,000]) [45]. The total mean annual costs for previous stroke among people with T2D in Sweden was more similar to our estimate at €11,397 [20]. This reflected a cost that was 2.2 times higher than that of people without T2D (€5797) and the costs associated with stroke were greater than the impact of atherosclerotic cardiovascular disease generally [20]. A previous study conducted in the French SNDS reported a mean global reimbursed expenditure of €5500 in 2015 for overall T2D patients [46]. The mean total direct medical cost of €18,592 reported in our current study for people with T2D and stroke aligns with the findings from a systematic review describing a median annual cost per patient for cardiovascular disease 3-times higher than in those for people with T2D without cardiovascular disease [47].

Taken together, these findings emphasize the need for a strong and rigorous primary and secondary prevention of cardiovascular diseases such as stroke in the T2D population.

### Strengths and limitations

A major strength of the present study was the use of a large, nationally representative sample of people with T2D and incident stroke. The breadth of the EGB database enabled a comprehensive assessment of the clinical and economic burden of stroke in people with T2D. In particular, the use of this database enabled the comprehensive description of direct medical costs and HCRU including outpatient visits. Despite the breadth of the data, data for key clinical variables including glycated hemoglobin, lipid profiles and blood pressure measurements were unavailable for use to explore the potential explanations for the observed treatment patterns at baseline and following incident stroke. A further limitation of the study was that estimates of stroke recurrence are likely to reflect an underestimate of the true stroke recurrence rate. This is since stroke recurrences occurring within 21 days of index date were excluded from the estimates due to difficulties associated with disentangling separate stroke events using administrative databases [22]. Given that the risk of stroke recurrence is greatest in the immediate poststroke period, a considerable number of recurrent strokes are likely to have been excluded [48].

Another important limitation of this study was that ICD-10 codes were used to identify patient comorbidities and clinical outcomes (e.g., cardiac arrythmias) and, in some cases, may not represent the true prevalence and incidence of these conditions. However, previous studies have shown that ICD-10 codes accurately identify patients with cardiovascular disease such as heart failure [49]. Our study also lacks data regarding intravenous thrombolysis and endovascular treatment reperfusion procedures, or any conservative or minimal invasive surgery. Lastly, these data reflect outcomes and treatments for the period between 2012 and 2019, prior to the availability of SGLT2i in France and clinical guideline updates. As such, these data may not reflect current clinical practice trends. Additional studies are required to explore the impact of the widespread availability of SGLT2i on the outcomes of people with T2D with prior stroke.

## Conclusions

The incidence of stroke is high among people with T2D, with ischemic stroke being the most common presentation. Stroke is associated with considerable clinical and economic burden among people with T2D, yet only a low proportion of people living with T2D receive recommended cardiovascular risk reducing therapies as per clinical guidelines. These findings highlight the need for the strengthened and multidisciplinary approach for the management of cardiovascular disease in the T2D population.

### Supplementary Information

Table S1. Definition of clinical variables. Figure S1. Study design. Figure S2. Flow chart. Figure S3. Time to first cardiovascular event according to incident stroke subtype. Supplementary Material 1

## Data Availability

Data were shared by the French national health insurance: ‘Caisse Nationale d’Assurance Maladie’ (CNAM).

## Abbreviations

ADA: American Diabetes Association
CHD: Coronary heart disease
CI: Confidence interval
CNAM: Caisse Nationale d’Assurance Maladie
DPP-4: Dipeptidyl peptidase-4
EASD: European Association for the Study of Diabetes
EGB: Echantillon Général des Bénéficiaires
ESC: European Society of Cardiology
GLP-1: Glucagon-like peptide 1 receptor
HCRU: Health Care Resource Utilization
ICD-10: International Classification of Disease, version 10
ICU: Intensive care unit
IQR: Interquartile range
PAD: Peripheral arterial disease
RA: Receptor agonist
RWE: Real-world evidence
SD: Standard deviation
SGLT2i: Sodium-glucose-like cotransporter 2 inhibitor
SNDS: French Système National des Données de Santé
T2D: Type 2 diabetes
