# Supplementary material for: Incidence of stroke, subsequent clinical outcomes and health care resource utilization in people with type 2 diabetes: a real-world database study in France: “INSIST” study
Source: Cardiovasc Diabetol. 2024 May 29;23:183. doi: 10.1186/s12933-024-02257-4 (PMC11137927; doi:10.1186/s12933-024-02257-4)
Supplement: Supplementary file 1 — Table S1. Definition of clinical variables. Figure S1. Study design. Figure S2. Flow chart. Figure S3. Time to first cardiovascular event according to incident stroke subtype. Supplementary Material 1 [file 12933_2024_2257_MOESM1_ESM.pdf]

Supplementary Materials: Incidence of Stroke, Subsequent Clinical Outcomes and Health Care Resource Utilization in People with Type 2 Diabetes: A Real-World Database Study in France - "INSIST" Study

Kamel Mohammedi[1], MD, PhD; Laurent Fauchier[2], MD, PhD; Nadia Quignot[3], PhD; Artak Khachatryan[4], MD, PhD; Tamar Banon[5], MSc; Raissa Kapnang[3], Engineer; Kazue Kikuchi[3], MPH; Hongye Ren[6], MPH; Christine Massien[7], MD; Lucile Vigié[7], MD; Sara Larsen[6], PhD; Igor Sibon[8], MD, PhD.

[1]INSERM unit 1034, Biology of Cardiovascular Diseases, Bordeaux University Hospital, F-33000, Bordeaux, France, [2]Cardiology Dept, Trousseau University Hospital, Tours, and University of Tours, France, [3]Evidence & Access, Certara France, Paris, France, [4]Evidence & Access, Certara UK, Sheffield, UK, [5]Evidence & Access, Certara Canada, Montreal, QC, Canada, [6]Novo Nordisk Denmark A/S, Copenhagen, Denmark, [7]Novo Nordisk France, Puteaux, France, [8]Bordeaux University Hospital, Stroke Unit, Bordeaux.

Corresponding author contact information:

Hôpital Haut-Lévêque

Department of Endocrinology, Diabetes, and Nutrition

Avenue de Magellan, 33604 PESSAC CEDEX

Phone: +33 5 57 65 60 78; Fax: +33 5 57 65 65 29

E-mail: km.mmohammedi@gmail.com

Contents

Additional file 1: Table S1. Definition of clinical variables 3

Additional file 1: Figure S1 Study design........ 10

Additional file 1: Figure S2. Flow chart 11

Additional file 1: Figure S3. Time to first cardiovascular event according to incident stroke subtype 12

Additional file 1: Table S1. Definition of clinical variables

Name	Type	Code	Description
All type of cerebrovascular disease	ICD-10	G45	Transient ischemic attacks and related syndromes
	ICD-10	G46	Cerebral vascular syndromes in cerebrovascular diseases
	ICD-10	G81	Hemiplegia
	ICD-10	I60	Subarachnoid hemorrhage
	ICD-10	I61	Intracerebral hemorrhage
	ICD-10	I62	Other non-traumatic intracranial hemorrhages
	ICD-10	I63	Cerebral infarction
	ICD-10	I64	Cerebrovascular accident, not specified as hemorrhagic or infarction
	ICD-10	I66	Occlusion and stenosis of cerebral arteries not leading to cere
	ICD-10	I67	Other cerebrovascular diseases
	ICD-10	I68	Cerebrovascular disorders in diseases classified elsewhere
	ICD-10	I69	Sequelae of cerebrovascular diseases
	DRG	01C044	Craniotomies without any trauma age over 17 years level 4
	DRG	01K071	Intracranial and spinal embolizations for hemorrhage level 1
	DRG	01K072	Intracranial and spinal cord embolizations for hemorrhage level 2
	DRG	01K073	Intracranial and spinal cord embolizations for hemorrhage level 3
	DRG	01K074	Intracranial and spinal cord embolizations for hemorrhage level 4
	DRG	01M101	Other cerebrovascular conditions level 1
	DRG	01M102	Other cerebrovascular conditions level 2
	DRG	01M103	Other cerebrovascular conditions level 3
	DRG	01M104	Other cerebrovascular conditions level 4
	DRG	01M10T	Other cerebrovascular conditions very short duration
	DRG	01M163	Transient ischemic attacks and occlusions of precerebral arteries, age less than 80 years, level 3
	DRG	01M301	Non-transient intracerebral vascular accidents level 1
	DRG	01M302	Non-transient intracerebral vascular accidents level 2
	DRG	01M303	Non-transient intracerebral vascular accidents level 3
	DRG	01M304	Non-transient intracerebral vascular accidents level 4
	DRG	01M30T	Transfers and other short stays for non-transient intracerebral
	DRG	01M311	Other non-transient strokes level 1
	DRG	01M312	Other non-transient strokes level 2
	DRG	01M313	Other non-transient strokes level 3
	DRG	01M314	Other non-transient strokes level 4
	DRG	01M31T	Transfers and other short stays for other non-transient strokes
	DRG	01M36E	Non-transient cerebrovascular accidents with death: stays of le
Aortic disease	ICD-10	I06	Rheumatic diseases of the aortic valve
	ICD-10	I080	Damage to the mitral and aortic valves
	ICD-10	I082	Damage to the aortic and tricuspid valves
	ICD-10	I083	Damage to the mitral, aortic and tricuspid valves
	ICD-10	I359	Damage to the aortic valve, unspecified
	ICD-10	I700	Atherosclerosis of the aorta
	ICD-10	I71	Aortic aneurysm and dissection
	ICD-10	I740	Embolism and thrombosis of the abdominal aorta
	ICD-10	I749	Embolism and thrombosis of unspecified arteries

	ICD-10	I790	Aortic aneurysm in diseases classified elsewhere
	CCAM	DGAF005	Intraluminal dilatation of the abdominal aorta without stent placement, by transcutaneous arterial route
	CCAM	DGAF008	Intraluminal dilatation of the abdominal aorta without stent placement, by transcutaneous arterial route
	CCAM	DGFA001	Thromboendarterectomy of the abdominal aortic trunk, by laparotomy
	CCAM	DGFA003	Thromboendarterectomy aortobisiliac, by laparotomy
	CCAM	DGFA004	Thrombectomy of the abdominal aorta, common iliac artery and/or external iliac artery, via bilateral inguinofemoral approach
	CCAM	DGFA005	Thrombectomy of a bypass or thromboendarterectomy of the abdominal aorta or its branches, by laparotomy
	CCAM	DGFA007	Thromboendarterectomy unilateral aorto-ilio-femoral, by laparotomy
	CCAM	DGFA008	Bilateral aorto-ilio-femoral thromboendarterectomy, by laparotomy
	CCAM	DGFA009	Unilateral aortoiliac thromboendarterectomy, by laparotomy
	CCAM	DGFA010	Thrombectomy of the abdominal aorta, common iliac artery and/or external iliac artery, by laparotomy
	CCAM	DGFA011	Thromboendarterectomy of the trunk and visceral branch of the juxta diaphragmatic aorta, by thoraco-phreno-laparotomy without CEC
	CCAM	DGFA012	Thrombectomy of a bypass or thromboendarterectomy of the abdominal aorta or its branches, by inguinofemoral approach
	CCAM	DGLF001	Placement of covered bifurcated aortobisiliac stent, via transcutaneous arterial route
	CCAM	DGLF002	Placement of rectilinear covered stent in the infrarenal abdominal aorta, by transcutaneous arterial route
	CCAM	DGLF005	Placement of fenestrated or multibranch endoprosthesis in the abdominal aorta for complex aneurysm, by transcutaneous arterial route
	CCAM	DGLF012	Flattening of an iliac aneurysm with unilateral ilio-iliac or iliofemoral prosthetic replacement, by laparotomy
	CCAM	DGPA001	Flattening of a ruptured infrarenal or aortobisiliac aortic aneurysm with prosthetic replacement, by laparotomy
	CCAM	DGPA005	Flattening of an unruptured infrarenal aortic aneurysm with prosthetic aortoaortic replacement infrarenal, by laparotomy with suprarenal clamping
	CCAM	DGPA008	Flattening of an unruptured infrarenal aortic aneurysm with prosthetic aortoaortic replacement infrarenal, by laparotomy with infrarenal clamping
	CCAM	DGPA010	Flattening of an unruptured infrarenal or aortobisiliac aortic aneurysm with aortobifemoral prosthetic replacement, by laparotomy with suprarenal clamping
	CCAM	DGPA012	Flattening of an unruptured infrarenal or aortobisiliac aortic aneurysm with aortobisiliac prosthetic replacement, by laparotomy with suprarenal clamping
	CCAM	DGPA013	Flattening of an unruptured infrarenal or aortobisiliac aortic aneurysm with aortobisiliac prosthetic replacement, by laparotomy with infrarenal clamping
	CCAM	DGPA016	Flattening of an aorto -ilio-femoral aneurysm with bifurcated aorto -ilio-femoral prosthetic replacement, by laparotomy with suprarenal clamping

	CCAM	DGPA017	Flattening of an unruptured infrarenal aortic aneurysm with infrarenal aortoaortic prosthetic replacement, by laparotomy with suprarenal clamping
	CCAM	DGPA018	Flattening of an unruptured infrarenal or aortobisiliac aortic aneurysm with aortobifemoral prosthetic replacement, by laparotomy with infrarenal clamping
	CCAM	DGPF001	Intraluminal dilatation of the abdominal aorta with stent placement, by transcutaneous arterial route
	CCAM	DGPF002	Clearing the aortic bifurcation, via transcutaneous arterial route
	CCAM	EDAF002	Recanalization of the aortic bifurcation with stent placement, via bilateral transcutaneous arterial route
	CCAM	EDAF003	Intraluminal dilation of the common iliac artery and/or the external iliac artery without stent placement, by transcutaneous arterial route
	CCAM	EDFA006	Thromboendarterectomy iliac, by laparotomy
	CCAM	EDFA007	Thromboendarterectomy iliofemoral, by laparotomy
	CCAM	EDLF004	Placement of covered stent in the common iliac artery and/or the external iliac artery, by transcutaneous arterial route
	CCAM	EDLF005	Placement of covered bifurcated aortobisiliac stent, via transcutaneous arterial route
	CCAM	EDPA001	Flattening of an iliac aneurysm with unilateral aorto-iliac or aortofemoral prosthetic replacement, by laparotomy
	CCAM	EDPA005	Flattening of an iliac aneurysm with unilateral aortoiliac or aortofemoral prosthetic replacement, by laparotomy
	CCAM	EDPF008	Recanalization of the common iliac artery and/or the external iliac artery without stent placement, by transcutaneous arterial route
	CCAM	EDPF009	Recanalization of the common iliac artery and/or the external iliac artery with stent placement, via transcutaneous arterial route
Cardiac arrhythmias	ICD-10	I471	Supraventricular tachycardia
	ICD-10	I472	Ventricular tachycardia
	ICD-10	I48	Atrial fibrillation and flutter
	ICD-10	I49	Other cardiac arrhythmias
	ICD-10	P291	Neonatal cardiac arrhythmia
	CCAM	DEPF004	Complete interruption of several accessory pathways of cardiac conduction with radiofrequency current, through the transcutaneous vascular route
	CCAM	DEPF005	Complete interruption of an accessory pathway of cardiac conduction with radiofrequency current, through the transcutaneous vascular route
	CCAM	DEPF006	Complete interruption of the nodal tissue of the atrioventricular junction with radiofrequency current, through a transcutaneous venous route
	CCAM	DEPF010	Interruption of the slow pathway or the rapid pathway of the atrioventricular junction with radiofrequency current or cryotherapy, by transcutaneous venous route
	CCAM	DEPF012	Complete interruption of the arrhythmogenic circuit at the level of the cavotricuspid isthmus with radiofrequency current or cryotherapy, by transcutaneous venous route
	CCAM	DEPF014	Complete interruption of arrhythmogenic circuit outside the cavotricuspid isthmus with radiofrequency current, by transcutaneous

			venous route and transseptal route
	CCAM	DEPF025	Complete interruption of arrhythmogenic circuit outside the cavotricuspid isthmus with radiofrequency current, by transcutaneous venous route
	CCAM	DEPF033	Complete interruption of the arrhythmogenic circuit at the level of the ostium of the pulmonary veins with radiofrequency current or cryotherapy, by transcutaneous venous route and transseptal route
	CCAM	DEQF001	Cardiac electrophysiological exploration by right intracavitary probe by transcutaneous venous route, with provocative maneuvers of tachycardia at the ventricular level
	CCAM	DEQF002	Cardiac electrophysiological exploration by right intracavitary probe by transcutaneous venous route, with provocative maneuvers of tachycardia at the atrial level and at the ventricular level
	CCAM	DEQF003	Cardiac electrophysiological exploration by right intracavitary probe, by transcutaneous venous route
	CCAM	DEQF004	Cardiac electrophysiological exploration by right intracavitary probe by transcutaneous venous route, with provocative maneuvers of tachycardia at the atrial level
	CCAM	DEQF005	Cardiac electrophysiological exploration by right and left intracavitary probes by transcutaneous vascular route, with provocative maneuvers of tachycardia
	CCAM	DERF001	Low-energy cardiac electric shock, through the transcutaneous vascular route
	CCAM	DERF002	High energy cardiac electric shock, by transcutaneous vascular route
	CCAM	DERF003	Temporary electrical stimulation of the atrium to interrupt a supraventricular tachycardia, by transcutaneous venous route
	CCAM	DERP003	Transcutaneous Cardiac Electric Shock [External Cardioversion], outside of the ER
	CCAM	DERP004	Transcutaneous cardiac electric shock [External cardioversion], emergency
	CCAM	PAIN001	Temporary electrical stimulation of the atrium by the esophageal route, to interrupt a supraventricular tachycardia
	DRG	05M081	Arrhythmias and cardiac conduction disorders, level 1
	DRG	05M082	Arrhythmias and Cardiac Conduction Disorders, Level 2
	DRG	05M083	Arrhythmias and Cardiac Conduction Disorders, Grade 3
	DRG	05M084	Arrhythmias and Cardiac Conduction Disorders, Grade 4
	DRG	05M08T	Arrhythmias and cardiac conduction disorders, very short duration
	ATC	C01B	Antiarrhythmics, class I and III (for baseline characteristics definition only)
Carotid artery disease	ICD-10	I652	Carotid artery occlusion and stenosis
	CCAM	EBAF004	Intraluminal dilation of the cervical carotid artery without stenting, by the transcutaneous arterial approach
	CCAM	EBAF010	Intraluminal dilatation of the cervical carotid artery with stenting, transcutaneous arterial
	CCAM	EBCA001	Crossover carotidosubclavier or carotidoaxillary by cervicotomy
	CCAM	EBCA004	Cross-bridging, by cervicotomy
	CCAM	EBCA008	Carotidosubclaval or carotidaxillary ipsilateral bypass, by cervicotomy

	CCAM	EBCA015	Aortocardial, cervicotomy and thoracotomy bypass
	CCAM	EBCA017	Bridging between the common carotid artery and the ipsilateral internal carotid artery, by cervicotomy
	CCAM	EBEA003	Reimplantation of the subclavian artery into the common carotid artery by cervicotomy
	CCAM	EBEA005	Reimplantation of the common carotid artery into the subclavian artery, by cervicotomy
	CCAM	EBFA002	Thromboendarterectomy of the carotid bifurcation with widening angioplasty, by cervicotomy with vascular diversion
	CCAM	EBFA003	Thromboendarterectomy of the common carotid artery by cervicotomy
	CCAM	EBFA005	Thrombectomy of the common carotid artery by cervicotomy
	CCAM	EBFA006	Thromboendarterectomy of the carotid bifurcation without widening angioplasty, by cervicotomy with vascular diversion
	CCAM	EBFA008	Thromboendarterectomy of the carotid bifurcation by reversal, by cervicotomy without vascular diversion
	CCAM	EBFA010	Thromboendarterectomy of the common carotid artery by cervicotomy and thoracotomy
	CCAM	EBFA012	Thromboendarterectomy of the carotid bifurcation without widening angioplasty, by cervicotomy without vascular diversion
	CCAM	EBFA015	Thromboendarterectomy of the carotid bifurcation by reversal, by cervicotomy with vascular diversion
	CCAM	EBFA016	Thromboendarterectomy of the carotid bifurcation with widening angioplasty, by cervicotomy without vascular diversion
	CCAM	EBKA001	Replacement of the common carotid artery by cervicotomy
	CCAM	EBKA003	Replacement of the common carotid artery by cervicotomy and thoracotomy
	DRG	01C061	Interventions on the precerebral vasculature, level 1
	DRG	01C062	Interventions on the precerebral vasculature, level 2
	DRG	01C063	Interventions on the precerebral vasculature, level 3
	DRG	01C064	Interventions on the precerebral vasculature, level 4
Coronary heart disease	ICD-10	I20	Angina pectoris
	ICD-10	I21	Acute myocardial infarction
	ICD-10	I22	Subsequent myocardial infarction
	ICD-10	I23	Some recent complications of acute myocardial infarction
	ICD-10	I24	Other acute ischemic heart diseases
	ICD-10	I25	Chronic ischemic heart disease
	CCAM	DDMA003	Coronary revascularization by 3 arterial grafts with 3 distal anastomoses, by thoracotomy with CEC
	CCAM	DDMA005	Coronary revascularization by 3 arterial grafts with 3 distal anastomoses, by thoracotomy with CEC
	CCAM	DDMA006	Coronary revascularization by 3 arterial grafts with 3 distal anastomoses, by thoracotomy with CEC
	CCAM	DDMA008	Coronary revascularization by 3 arterial grafts with 3 distal anastomoses, by thoracotomy with CEC
	CCAM	DDMA011	Coronary revascularization by 3 arterial grafts with 3 distal anastomoses, by thoracotomy with CEC
	CCAM	DDMA012	Coronary revascularization by 3 arterial grafts with 3 distal

			anastomoses, by thoracotomy with CEC
Deep Vein Thrombosis	ICD-10	I801	Phlebitis and thrombophlebitis of the femoral vein
	ICD-10	I802	Phlebitis and thrombophlebitis of other deep vessels
	ICD-10	I803	Phlebitis and thrombophlebitis of lower limbs, unspecified
	ICD-10	I809	Phlebitis and thrombophlebitis of unspecified location
	ICD-10	I821	Other venous embolisms and thromboses
	ICD-10	I822	Embolism and thrombosis of the vena cava
	ICD-10	I828	Embolism and thrombosis of other specified veins
	ICD-10	I829	Embolism and thrombosis of an unspecified vein
	CCAM	DHQH001	Phlebography selective of several branches of the veins common iliac bones and/ or the inferior vena cava , via venous transcutaneous
	CCAM	DHQH002	Venography of the inferior vena cava [Cavography lower], by way venous transcutaneous
	CCAM	DHQH003	Venography of the superior vena cava [Superior cavography], by intravenous injection transcutaneous
	CCAM	DHQH004	Phlebography selective of a branch of the vein common iliac or inferior vena cava , via venous transcutaneous
	CCAM	DHQH005	Venography of the veins iliac and inferior cava [Iliocavography], by intravenous injection transcutaneous femoral unilateral Or bilateral
	CCAM	DHQH007	Phlebography hyperselective of a branch of the vein common iliac or inferior vena cava , via venous transcutaneous
	CCAM	DHQM002	ultrasound of the inferior vena cava and its tributaries
	CCAM	EFQM001	Doppler ultrasound of limb veins superiors
	CCAM	EJQM001	Doppler ultrasound of limb veins lower , with marking cutaneous Or cartography hemodynamic
	CCAM	EJQM003	Ultrasound-Doppler of the veins of the lower limbs and the iliac veins, to search for deep vein thrombosis
	CCAM	EJQM004	Doppler ultrasound of limb veins inferior and veins iliac, without marking cutaneous
Heart failure	ICD-10	I110	Hypertensive heart disease, with heart failure
	ICD-10	I50	Heart failure
Peripheral arterial disease	ICD-10	I702	Atherosclerosis of distal arteries
	ICD-10	I738	Other specified peripheral vascular diseases
	ICD-10	I739	Peripheral vascular disease, unspecified
	ICD-10	I73	Other peripheral vascular diseases
	ICD-10	I742	Embolism and thrombosis of the arteries of the upper limbs
	ICD-10	I743	Embolism and thrombosis of the arteries of the lower limbs
	ICD-10	I744	Embolism and thrombosis of distal arteries, unspecified
	ICD-10	I77	Other damage to the arteries and arterioles
	ICD-10	I79	Damage to arteries, arterioles and capillaries in diseases classified elsewhere
Pulmonary embolism	ICD-10	I260	Pulmonary embolism, with mention of acute cor pulmonale
	ICD-10	I269	Pulmonary embolism
	CCAM	DFQH001	Arteriography selective of the trunk and/ or branches of the artery pulmonary , by way venous transcutaneous
	CCAM	DHQH001	Phlebography selective of several branches of the veins common iliac

			bones and/ or the inferior vena cava , via venous transcutaneous
	CCAM	DHQH002	Venography of the inferior vena cava [Cavography lower], by way venous transcutaneous
	CCAM	DHQH003	Venography of the superior vena cava [Superior cavography], by intravenous injection transcutaneous
	CCAM	DHQH004	Phlebography selective of a branch of the vein common iliac or inferior vena cava , via venous transcutaneous
	CCAM	DHQH005	Venography of the veins iliac and inferior cava [Iliocavography], by intravenous injection transcutaneous femoral unilateral Or bilateral
	CCAM	DHQH007	Phlebography hyperselective of a branch of the vein common iliac or inferior vena cava , via venous transcutaneous
	CCAM	DHQM002	ultrasound of the inferior vena cava and its tributaries
	CCAM	ECQH010	Scanning of the vessels of the thorax and/ or heart [Angioscanner thoracic]
	CCAM	ECQH011	Scan of the vessels of the thorax and/ or heart , with scan of the vessels of the abdomen and/ or small pelvis [Angioscanner chest with CT angiography of the abdomen and/ or pelvis]
	CCAM	EFQM001	Doppler ultrasound of limb veins superiors
	CCAM	EJQM001	Doppler ultrasound of limb veins lower , with marking cutaneous Or cartography hemodynamic
	CCAM	EJQM003	Doppler ultrasound of limb veins inferior and veins iliacs , for detection of deep vein thrombosis
	CCAM	EJQM004	Doppler ultrasound of limb veins inferior and veins iliac , without marking cutaneous
	CCAM	EKQH001	Scan of limb vessels superiors [CT angiogram of the limbs superiors]
	CCAM	EMQH001	Scan of limb vessels lower [CT angiography of the limbs lower]
	CCAM	ZBQH001	CT scan of the chest, with intravenous injection of contrast product

CCAM: Classification Commune des Actes Médicaux, DRG: diagnosis-related group, ICD-10: International Classification of Diseases, 10th Revision

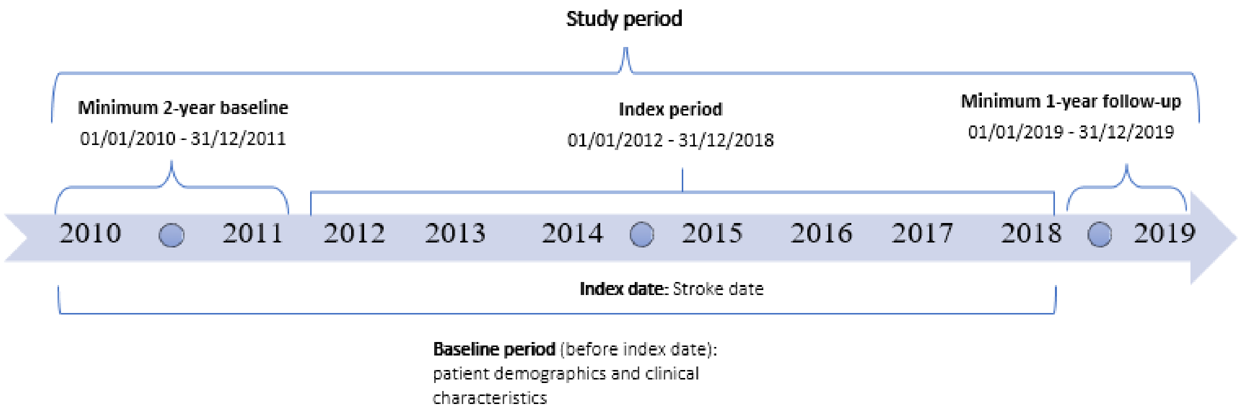

Additional file 1: Figure S1 Study design

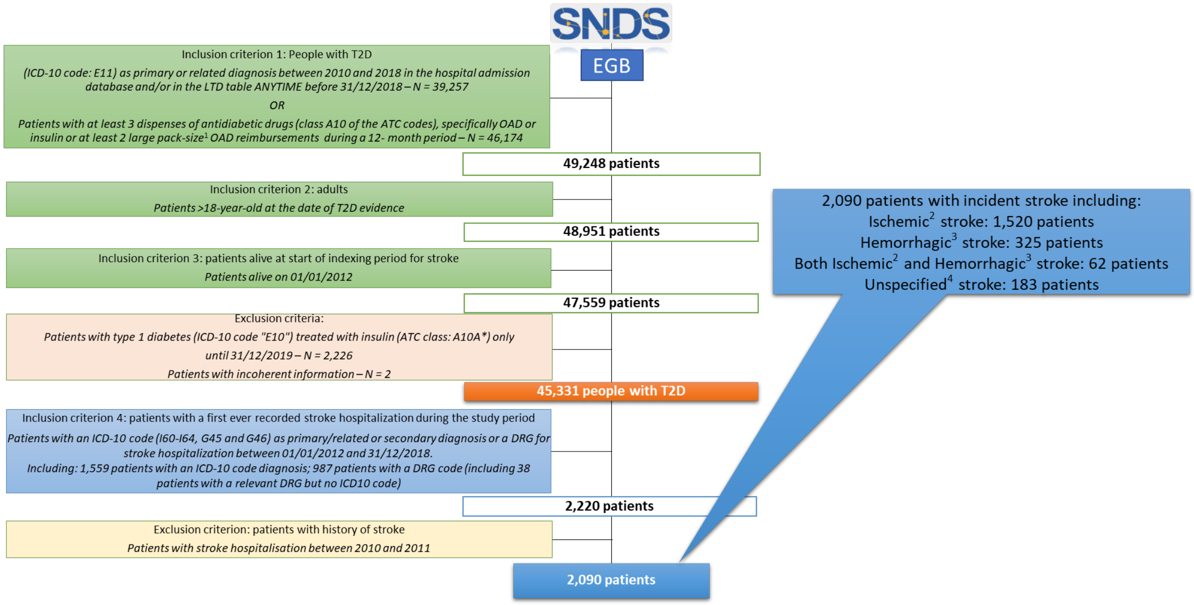

Additional file 1: Figure S2. Flow chart

ATC: Anatomical Therapeutic Chemical code, ICD-10: International Classification of Diseases, 10th Revision, LTD: long term disease, OAD: oral antidiabetic drug, T2D: type 2 diabetes,

[1]The large pack-size is defined as more than 90 capsules or tablets in one pack

[2] Ischemic: including ischemic, transient ischemic attack, and brain stem stroke (I63, G45 and G46 with I63 as secondary diagnosis)

Note that Ischemic stroke includes patients with both ischemic and hemorrhagic stroke codes on the same date (n=46)

[3] Hemorrhagic: including hemorrhagic and brain stem with hemorrhage (I60-I62 and G46 with I60-I62 as secondary diagnosis)

[4] Not specified: Stroke, not specified (I64 and G46 and I64 as secondary diagnosis)

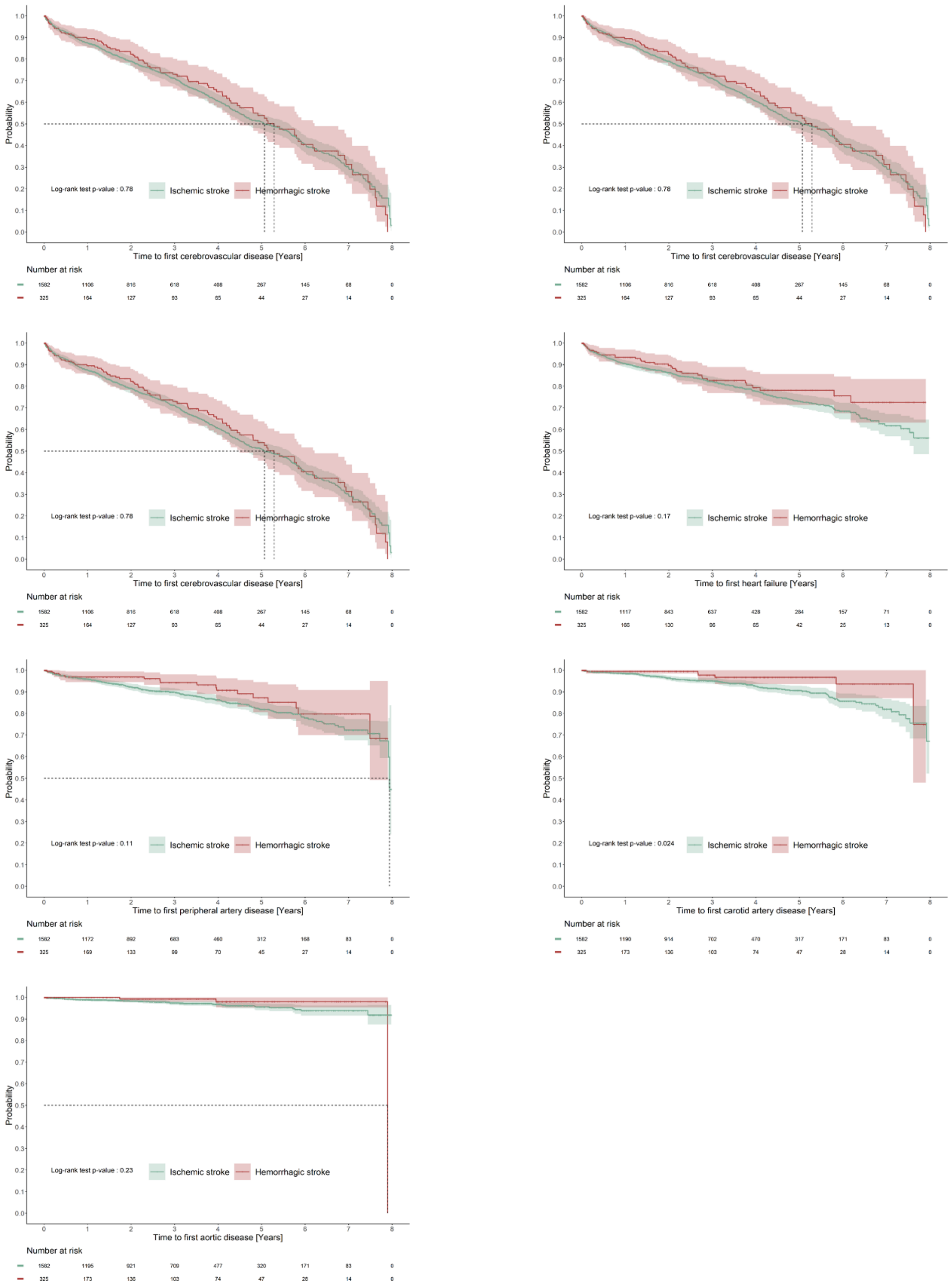

Additional file 1: Figure S3. Time to first cardiovascular event according to incident stroke subtype
